# Predicting the Impact of Alternative Splicing on Plant MADS Domain Protein Function

**DOI:** 10.1371/journal.pone.0030524

**Published:** 2012-01-25

**Authors:** Edouard I. Severing, Aalt D. J. van Dijk, Giuseppa Morabito, Jacqueline Busscher-Lange, Richard G. H. Immink, Roeland C. H. J. van Ham

**Affiliations:** 1 Applied Bioinformatics, Plant Research International, Wageningen, The Netherlands; 2 Laboratory of Bioinformatics, Wageningen University, Wageningen, The Netherlands; 3 Centre for BioSystems Genomics, Wageningen, The Netherlands; 4 Plant Developmental Systems, Plant Research International, Wageningen, The Netherlands; The Centre for Research and Technology, Hellas, Greece

## Abstract

Several genome-wide studies demonstrated that alternative splicing (AS) significantly increases the transcriptome complexity in plants. However, the impact of AS on the functional diversity of proteins is difficult to assess using genome-wide approaches. The availability of detailed sequence annotations for specific genes and gene families allows for a more detailed assessment of the potential effect of AS on their function. One example is the plant MADS-domain transcription factor family, members of which interact to form protein complexes that function in transcription regulation. Here, we perform an *in silico* analysis of the potential impact of AS on the protein-protein interaction capabilities of MIKC-type MADS-domain proteins. We first confirmed the expression of transcript isoforms resulting from predicted AS events. Expressed transcript isoforms were considered functional if they were likely to be translated and if their corresponding AS events either had an effect on predicted dimerisation motifs or occurred in regions known to be involved in multimeric complex formation, or otherwise, if their effect was conserved in different species. Nine out of twelve MIKC MADS-box genes predicted to produce multiple protein isoforms harbored putative functional AS events according to those criteria. AS events with conserved effects were only found at the borders of or within the K-box domain. We illustrate how AS can contribute to the evolution of interaction networks through an example of selective inclusion of a recently evolved interaction motif in the MADS AFFECTING FLOWERING1-3 (MAF1–3) subclade. Furthermore, we demonstrate the potential effect of an AS event in SHORT VEGETATIVE PHASE (SVP), resulting in the deletion of a short sequence stretch including a predicted interaction motif, by overexpression of the fully spliced and the alternatively spliced *SVP* transcripts. For most of the AS events we were able to formulate hypotheses about the potential impact on the interaction capabilities of the encoded MIKC proteins.

## Introduction

Alternative splicing (AS) is a frequent phenomenon in higher eukaryotes that involves the production of multiple distinct transcript isoforms from a single gene. Genome-wide studies have shown that the pre-mRNAs of around 40% of plant genes are alternatively spliced [1]. One of the roles that is ascribed to AS is that of a mechanism for controlling gene expression at the post transcriptional level [2]. The second role is that of a mechanism for increasing protein diversity [3]. However, the extent to which this increased protein diversity is functional is not well known.

Several genome-wide studies have addressed this issue by determining the prevalence of AS events that are likely to be functional according to predefined criteria such as conservation [4] or the predicted effect on protein structure [5], [6], [7]. Other genome-wide studies focused on the identification of more general patterns that relate AS to gene or domain functions [8], [9], and although a number of interesting patterns has been unveiled, by their design, these studies only identify aspects that are general enough to be present in large numbers of proteins. However, each gene and gene family has its own evolutionary history and can be affected by AS in specific ways that cannot be described by globally observed patterns. The way in which a gene is affected by AS depends for instance on the specific genomic rearrangements, such as tandem exon duplications that have occurred in the gene's evolutionary history [10], [11]. Hence, in order to fully value the functional impact of AS, it is important to also study the process at the level of individual genes or gene families.

One of the best studied gene families in plants is the MADS-box transcription factor family. Members of this family are involved in a number of developmental processes [12] but they are probably best known for their role in regulating the onset and patterning of flowering [13]. MADS-box genes can be divided into two main groups: the type I and type II or MIKC classes [14], [15]. While little is known about the former group, a wealth of information is available for the latter. MIKC proteins mainly exert their function in the form of di- or multimeric protein complexes [16]. The availability of a comprehensive yeast two-hybrid interaction map for *Arabidopsis thaliana* MADS-domain proteins [17] as well as the results of an extensive yeast three-hybrid screen [18] illustrate the large diversity of complexes that are potentially formed between members of this family.

The sequence of MIKC proteins can be divided into four regions with specific functions [19], [20]. The MADS (M) domain has a DNA-binding function and, together with the intervening (I) domain, is involved in determining the specificity of protein dimerization. The dimeric protein-protein interaction is promoted by the Keratin-like (K) domain. This region of the protein is supposed to fold into three consecutive amphipathic α-helices, referred to as K1, K2 and K3, from which the first two helices are important for dimerization [16], [21]. For K3 a role in higher-order complex formation has been demonstrated [22]. The C-terminal (C) domain is involved in transcriptional activation or repression [23] and furthermore, in the formation of higher-order complexes [16]. Recently, we developed computational methods that aid in the identification and understanding of sequence features that are important determinants of the interaction specificity of individual MIKC proteins [24], [25].

One of the ways through which AS can influence the formation of di- or multimeric complexes is by regulating the availability of individual proteins. This can be achieved by regulating the production of transcripts that are targets for the Nonsense Mediated Decay (NMD) pathway [2]. Alternatively, AS can influence the interaction specificity of the encoded proteins by disrupting or introducing individual interaction sites. This has for instance previously been shown for the two AS variants of the *Arabidopsis B-sister (ABS, AGL32)* gene (a member of the MIKC family) that encode proteins with different higher-order interaction specificities and functions [18], [26], [27], [28]. Another example involves the members of the myocyte enhancer factor 2 (MEF2) MADS subfamily in humans which can form over 100 different dimers through AS [29]. On the functional level, AS mediated changes to binding properties can result in functional antagonists as demonstrated for other classes of proteins, such as e.g. the TRα-1/c-erbAα-2 system in rats (reviewed in [30]). Whether this type of antagonistic functional diversification through AS occurs within the plant MADS domain transcription factor family as well, is still unknown.

Several individual cases of AS events have previously been reported for members of the MADS-box gene family (e.g. [26], [28], [31], [32], [33], [34], [35], [36], [37], [38], [39], [40], [41]). However, a systematic analysis of the functional impact of AS within this family is still lacking. In this study we investigate the potential impact of identified AS events on the formation of protein complexes between MIKC proteins. We analyzed the impact of AS on both the MIKC-type protein group as a whole and on a selection of individual members. Three independent criteria were used to postulate a functional implication of an AS event. The first criterion required AS to overlap with predicted dimerisation interaction motifs that can be considered to correspond to those regions that form the contact surface, using our recently developed interaction motif prediction method [24], [25]. The second criterion required overlap with the region known to be important for higher-order complex formation. In contrast to these criteria, which focus on the nature of the function, the third criterion considered conservation of an AS-induced polymorphism (AIP) at the protein level among different species and only indicates a potential function. In addition to providing interesting candidates for further experiments, our results illustrate the potential of computational analysis of AS within the functional context of individual genes or gene families.

## Results

### General data description and alternative splicing analysis

In total, 80 of the 111 annotated MADS-box genes (including MADS-box like) in *Arabidopsis* had at least one annotated protein for which the corresponding open reading frame was fully supported by transcript evidence (Table S1) and were considered further. Of the 38 loci for which at least one encoded protein had a clear MIKC structure, 31 had at least one annotated protein product with experimentally identified protein-protein interactions, and 17 had evidence for AS.

We first investigated the prevalence of AS within the DNA regions encoding for the individual domains of these MIKC proteins. To this end we clustered introns based on their genomic coordinates. Clusters containing multiple introns always corresponded to AS events. Singleton clusters on the other hand only corresponded to an AS event if the intron was found to be retained in one or more transcripts. The fractions of intron clusters that were involved in AS events were 24% (11/46), 11% (16/147) and 9.1% (2/22) for the I-, K-box- and C-terminal domain encoding DNA sequences, respectively. Fisher's exact tests indicated that AS was not significantly favored in any of the analyzed domains (best P-value: 0.03 for the I-domain). Nevertheless, intron clusters in the I-domain were twice as frequently involved in AS events than those located in the K-box or C-terminus domains. Given that the K-box domain is a PFAM domain and the I-domain is not, the lower fraction of AS within the K-box domain encoding DNA sequences is consistent with our previous observation that intron clusters within predicted PFAM-protein domain encoding DNA regions are less frequently involved in AS events than intron clusters located outside such predicted domains [4]. We did not find any AS event that affected the MADS domain. This is not surprising because there is only one *Arabidopsis* MADS box gene (*At1g33070*) that has introns in its MADS domain encoding sequence [42] and we did not find transcript (EST) support for this gene (Table S1).

AS can introduce premature termination codons (PTC) in transcripts, which may result in the production of truncated protein. However, many of these PTC containing transcripts are recognized by the cell and degraded via the NMD pathway [2]. Because in this study we are only interested in the function of AS events that are manifested at the protein level, all AS events resulting in putative NMD targets were discarded. Transcripts were regarded to be potential NMD targets if they contained a PTC that was located more than 55 nt upstream of the last exon/intron junction [43]. In a number of cases it was difficult to determine whether the transcript was likely to be degraded or translated from a downstream ATG codon due to the position of the PTC. After removal of these ambiguous cases, a total of twelve loci, accounting for 13 AS events, remained and were predicted to produce multiple protein isoforms (Table 1).

**Table 1:** Alternative spliced loci producing multiple protein isoforms.

TAIR 10 Locus	Symbol	Event type	Effect	Corresponding TAIR 10 identifiers
AT1G24260	*AGL9, SEP3*	alternative donor	**I-/K-box domain border:**1 AA difference	**AT1G24260.2** **AT1G24260.1**
AT1G77080	*FLM, AGL27, MAF1*	mutually exclusive exons	**I-domain:**Exon-5′: 19 AAExon-3′: 15 AA	**AT1G77080.2** **AT1G77080.4**
		cryptic exon	**C-terminus:**Addition of 10AA followed by a 33 AA truncation	**AT1G77080.4/AT1G77080.5**
		intron retention	**K-box**26 AA difference	**AT1G77080.2/EST-based**
AT2G22540	*AGL22, SVP*	alternative donor	**I-domain**4 AA difference	**AT2G22540.1/AT2G22540.2**
AT2G42830	*AGL5, SHP2*	alternative donor	**K-box:**2 AA difference	**AT2G42830.2/AT2G42830.1**
AT3G58780	*AGL1, SHP1*	alternative acceptor	**I-domain:**6 AA difference	**AT3G58780.1/AT3G58780.2**
AT4G09960	*AGL11, STK*	Exon Skipping	**K-box:**14 AA difference	**AT4G09960.1/AT4G09960.2**
AT5G23260	*AGL32, ABS, TT16*	alternative acceptor	**K-box:**5 AA difference	**AT5G23260.2/AT5G23260.1**
AT5G51860	*AGL72*	alternative acceptor	**C-terminus:**9 AA difference	**AT5G51860.1/AT5G51860.2**
AT5G65060	*AGL70, FCL3, MAF3*	alternative acceptor	**K-box:**10 AA difference	**AT5G65060.1/AT5G65060.2**
AT5G65050	*MAF2, AGL31*	alternative acceptor	**K-box:**10 AA difference	**AT5G65050.2/EST-based**
AT5G10140	*FLF, AGL25, FLC*	alternative acceptor	**C-terminus:**4AA insertion followed by 33 AA truncation	**AT5G10140.1/AT5G10140.2**
		exon skipping	**K-box/C-terminus:**14 AA difference	**AT5G10140.1/AT5G10140.4**
		alternative acceptor	**C-terminus:**9 AA Deletion followed by17 AA frame shift followed by6 AA deletion	**AT5G10140.1/EST-based**
		cryptic exon	**C terminus:**Insertion: 23 AA followed by 33 AA truncation	**AT5G10140.1/AT5G10140.3**
AT2G03710	*AGL3, SEP4*	alternative acceptor	**C-domain:**1 AA difference	**AT2G03710.1/AT2G03710.2**

The type of event and effect on the protein domain architecture is provided for each AS event that results in a translatable mRNA. For each event the TAIR 10 identifiers of the corresponding variants are given when possible. The EST-based annotation corresponds to variants that were not present in the TAIR 10 annotation.

### Experimental validation of predicted AS events

Before performing detailed bioinformatics analyses we investigated whether selected AS transcript isoforms are expressed at detectable levels. For this purpose, qRT-PCR experiments were performed starting with RNA isolated from tissues in which the fully spliced isoform of the corresponding gene is relatively strongly expressed. Using this approach, almost all AS events could be confirmed (Table 2) and in all these cases sequencing of the amplified fragment revealed the expected isoform. Subsequently, we searched for evidence of expression at the protein level by analyzing RNAseq data from Jiao and Meyerowitz [44], which represent fragments of flower-expressed RNA molecules that were associated with ribosomes (translatome). This data set provides evidence for translation of the transcript isoforms. Whether the produced proteins are stable cannot be deduced from this information. Using this dataset, we were able to confirm AS events from *FLM/MAF1*, *SVP* and *SEP3* (Table 3). Unfortunately, no translatome data was available for vegetative stages of development. Nevertheless, our analyses show expression at the mRNA level for the majority of cases and evidence for translation for a number of floral expressed AS isoforms.

**Table 2:** Additional expression evidence for the predicted functional AS event.

TAIR locus	Symbol	Tissue	Expression level^a^
AT5G08290	*YLS8* (Reference gene)	Leaf	17.2
AT5G08290	*YLS8* (Reference gene)	Carpel	17.1
AT2G22540	*SVP1/SVP3* b	Leaf	19.4
AT2G22540	*SVP3*	Leaf	3.2
AT1G77080	*MAF1.2*	Leaf	15.4
AT1G77080	*MAF1.4*	Leaf	16.1
AT5G65050	*MAF2.2*	Leaf	11.2
AT3G58780	*SHP1.1*	Carpel	10.7
AT3G58780	*SHP1.2*	Carpel	12.8
AT2G42830	*SHP2.1*	Carpel	9.9
AT2G42830	*SHP2.2*	Carpel	2.7
AT4G09960	*STK.1*	Carpel	14.6
AT4G09960	*STK.2*	Carpel	10.4
AT5G23260	*ABS.1*	Carpel	11.7
AT5G23260	*ABS.2*	Carpel	13.8
AT1G24260	*SEP3.1*	Carpel	17.0
AT1G24260	*SEP3.2*	Carpel	10.2

a The difference in cycle threshold (CT) value between the gene- or isoform-specific reaction and the non-template control reaction with the same oligonucleotides is given. Most of the “non-template” control reactions did not give any amplification after 40 cycles and in these cases a CT of 40 was taken for the calculation. Hence, the higher the number, the higher the expression level.

b No oligonucleotides could be designed that are specific for the *SVP1* splicing variant only. The indicated primers detect both *SVP1* and *SVP3.*

The tissue for which the expression analysis was performed is indicated. The selection of tissue is based on a relative high expression of the fully spliced transcripts in the respective tissues. Isoform specific oligonucleotide sets were used (see Table S3).

**Table 3:** Additional translatome evidence for the predicted functional AS events.

TAIR locus	Symbol	Splicing event	Translatome reads
AT1G77080	FLM/MAF1	mutually exclusive exons	88/55*MAF1.4/MAF1.2*
		intron retention	29/38*MAF1.4*/Retainedintron
AT2G22540	AGL22/SVP	alternative donor	48/1*SVP1*/*SVP3*
AT2G42830	AGL5/SHP-2	alternative donor	X
AT4G09960	AGL11/STK	exon skipping	X
AT5G23260	AGL32/ABS	alternative acceptor	X
AT5G65050	MAF2	alternative acceptor	X
AT5G65060	MAF3	alternative acceptor	X
AT1G24260	AGL9/SEP3	alternative donor	361/221*SEP-3.2/SEP-3.1*

The total numbers of reads supporting each of the alternatives are given for the translatome data as determined using MISO. “X” is used to denote that an AS event could not be confirmed in this analysis.

### Predicted impact of AS on protein interactions

**Figure 1:**
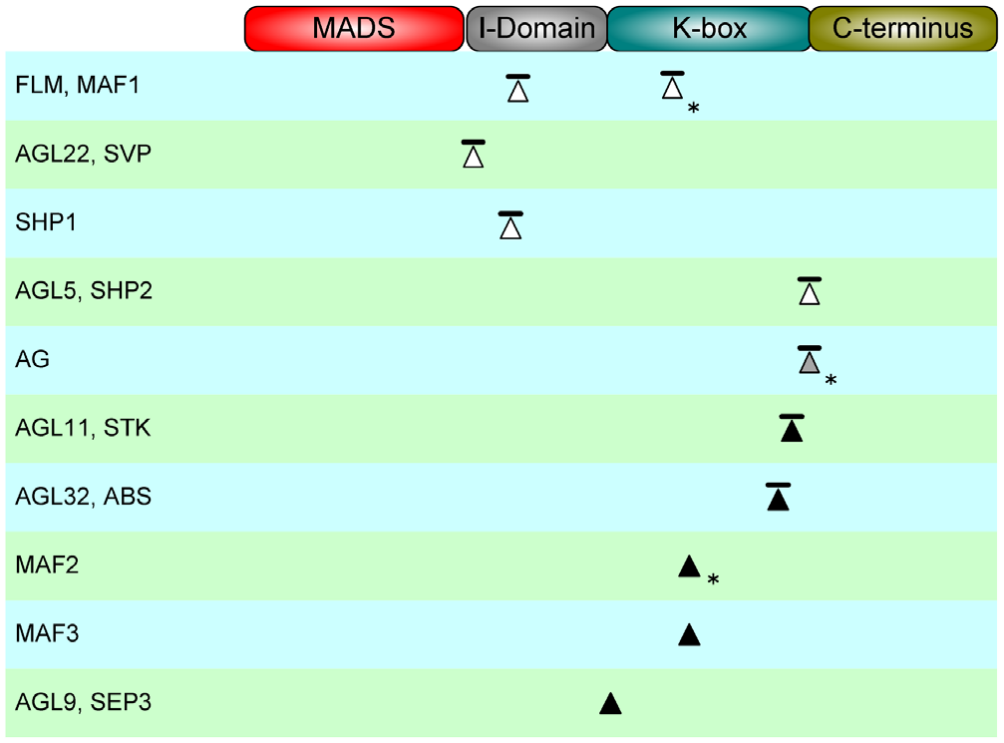
Putatively functional Alternative Splicing (AS) events in the *Arabidopsis* MIKC-type MADS-box family. Triangles indicate the position of AS events in relation to the protein-domain architecture of MIKC proteins. Bars on top of the triangles indicate that the event overlapped with at least one computationally predicted interaction motif. Asterisks indicate that the transcripts associated with the AS events are not annotated in TAIR 10. The color of a triangle indicates whether the AS induced polymorphism corresponding to the event was only identified in *Arabidopsis* (white), was conserved between different species but was not observed in *Arabidopsis* (grey) or, was conserved between *Arabidopsis* and other species (black).

The functional impact of AS was analyzed by investigating the effect of AS on predicted interaction motifs and on regions responsible for higher-order complex formation. The interaction motifs were predicted using our recently developed IMSS method [24], [25]. Ten out of the thirteen candidate events (nine of the twelve loci) were considered functional because they met at least one of the above criteria. All of these events were located in the I- or K-region of the proteins (Figure 1). Seven of the functional AS events (six loci) were found to overlap with sequences encoding for predicted interaction motifs (Figure 1: horizontal bars; Table S2). Three of these events overlapped with interaction motifs located in the I-domain, which was previously shown to be a hotspot for determination of interaction specificity [24]. The IMSS predictor was used to predict whether I-domain isoforms have different dimer interaction specificities. Only the isoforms of the *SHORT VEGETATIVE PHASE (SVP)* locus, which differ by the presence of a sequence containing a single interaction motif, were predicted to have different interaction specificities. The short SVP isoform (previously named SVP3 or SVP1 EFCSSS56-61D; [24]) lacking the motif was predicted to have five dimerization partners. The long SVP isoform (previously named SVP1; [24]) was predicted to have a total of 26 interaction partners. The large loss of predicted interaction partners for the short isoform corresponds well with the experimental yeast-based interaction studies [24].

Four AS events (four loci) were found to overlap with sequences encoding interaction motifs located within the K-box domain. Of these, only the AS event of the *MAF1* locus was located within the N-terminal region of the K-box which has been demonstrated to mediate dimer interactions [21]. This AS event involves the retention of an intron which leads to the introduction of an interaction motif without disruption of the downstream protein sequence. The remaining three events were located in the C-terminal region of the K-box domain which has been shown to be important for higher-order interactions [18]. The isoforms encoded by the *ABS* locus have experimentally been shown to form different higher-order complexes [18]. Strikingly, the positions of the AS-induced variation in *SEEDSTICK* (STK) and *SHATTERPROOF2* (SHP2) are quite similar to that in ABS, which leads us to predict that these also impact higher-order complex formation (see further discussion below). In addition, according to our IMSS predictions, ABS should not have different dimer interaction specificities for its two protein isoforms, and this has indeed been shown experimentally [26].

### *In planta* analysis of *SVP* isoform function

**Figure 2:**
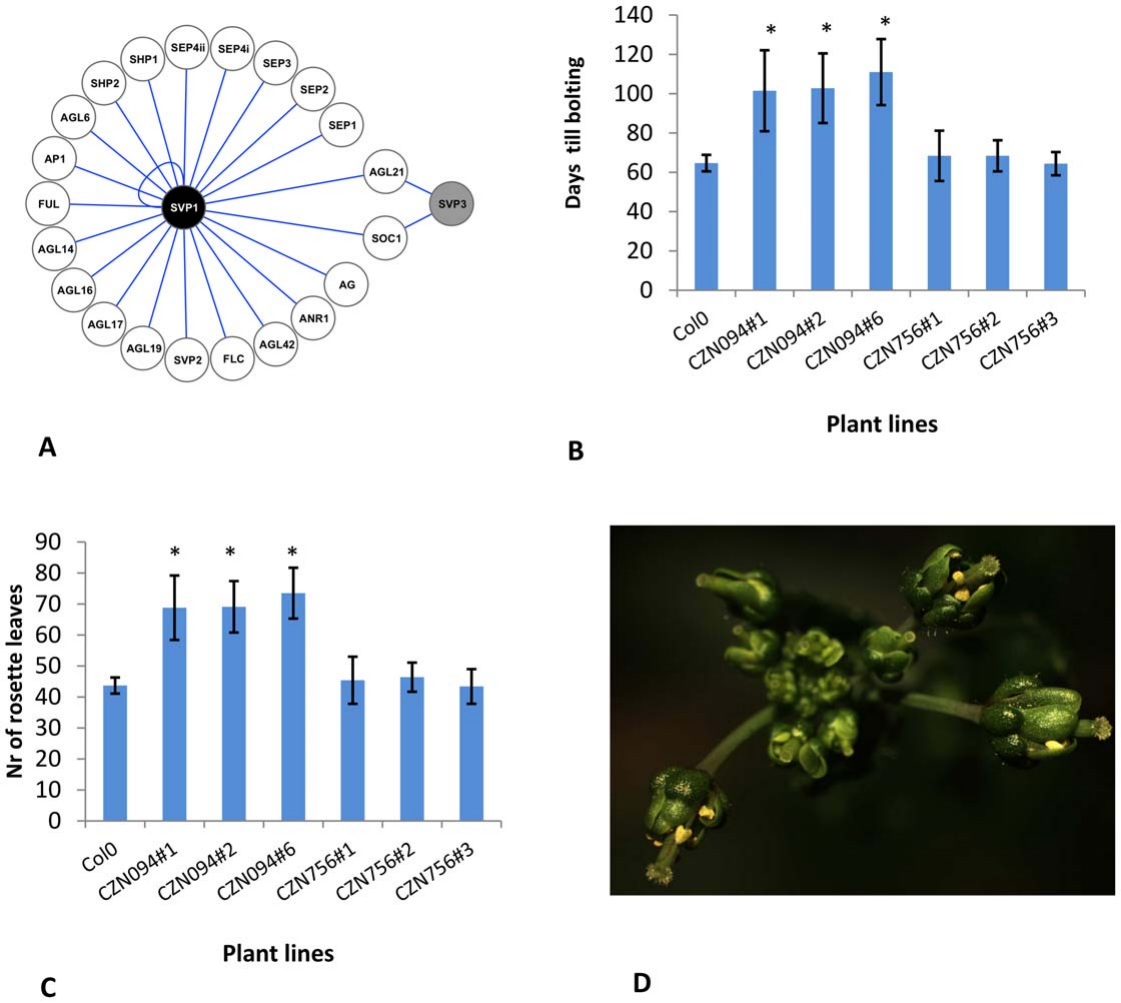
Functional analysis of two *SHORT VEGETATIVE PHASE (SVP)* isoforms. **A**) Graphical representation of protein-protein interaction capacity of the SVP1 (black) and SVP3 (grey) splicing variants as determined by matrix-based yeast two-hybrid studies (Van Dijk et al, 2010). **B**) and **C**) Effect of ectopic expression of *SVP1* (CZN094) and *SVP3* (CZN756) on flowering-time under short day conditions. Flowering-time was assessed using days until bolting (**B**) as well as the number of rosette leaves (**C**). For both constructs, flowering-time of three segregating lines was analyzed and compared to flowering-time of wild type control plants. * denotes statistical significance with p<0.01 (*t*-test). **D**) Floral phenotypes upon ectopic expression of *SVP1*. First and second whorl organs are greenish and leaf-like and flowers are partially sterile due to reduced anther filament elongation.

We performed overexpression studies in order to further substantiate the putative differences in the biological role of predicted AS isoforms. We selected *SVP* for this, because a strong effect of AS on dimerisation capacity of the encoded proteins is expected (Figure 2A) and hence on the putative function of the encoded protein isoforms. We performed overexpression studies with *SVP1* and *SVP3*, showing that ectopic expression of *SVP1* results in a late flowering phenotype and floral abnormalities (Figure 2B–D), as observed previously [45]. Occurrence of the floral abnormalities was linked to the strength of the floral repression in a segregating population. In contrast, ectopic expression of *SVP3* from the same *CaMV35S* promoter had no significant effect on flowering time (Figure 2B) and flowers developed without obvious modifications. Importantly, RT-PCR experiments confirmed ectopic expression of the *SVP3* transcript in the selected lines. Additional primary *CaMV35S::SVP3* transformants were selected and 15 were analyzed, but none showed obvious flowering time effects or floral abnormalities.

### Conservation of AS events

Next, we investigated for how many AS events the corresponding AIP was conserved in other plant species. Surprisingly, conserved AIPs were found for a total of six *Arabidopsis* MIKC loci (Figure 1, grey and black triangles), which is a rather high number given the limited of numbers events (<41 genes) conserved between orthologs that have been found in previous genome-wide analyses [4], [46], [47], [48]. The single-residue AIP located at the border of the I- and K-box domain encoding DNA sequence of *SEPALLATA3 (SEP3)* was detected in the closely related Brassicaceae species *Brassica napus* and *Raphanus sativus* (Figure S1). An additional AIP at this position involving two amino acid residues was conserved between the closely related Fabaceae species *Glycine max* and *Cyamopsis tetragonoloba*.

The recently duplicated *Arabidopsis MAF2* and *MAF3* paralogs have a conserved alternative acceptor event within the DNA sequence encoding for the N-terminal region of the K-box domain. The AIPs corresponding to these events were almost exactly conserved in both *Brassica napus* and *Brassica rapa* (Figure S2).

Three additional conserved AIPs were found that, in contrast to those of *SEP3* and *MAF2-3*, both overlapped with sequences encoding interaction motifs and that were located within the C-terminal region of the K-box domain (Figure 1). First, the AIP corresponding to the exon-skipping event of the *Arabidopsis STK* locus was also detected in the distantly related Asteraceae species *Taraxacum officinale* (Figure S3). Most likely, this represents a case of convergent evolution. Second, two different conserved AIPs were found between homologs of the *Arabidopsis ABS* locus. An AIP of the exact same size as the AIP of the *Arabidopsis ABS* locus was identified in *B. napus* (Figure S4). The second AIP was conserved between the species *Ricinus communis* and *Gossypium hirsutum* that belong to the Malvales and Balanopales orders, respectively. The stretch of residues involved in these AIPs is homologous to the entire exon that is affected by the AS event in the *Arabidopsis ABS* locus.

**Figure 3:**
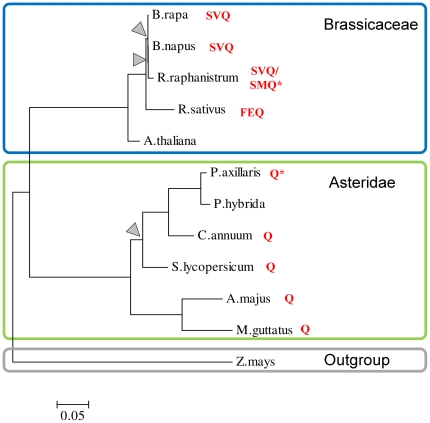
Conservation of AS induced polymorphisms (AIPs) between homologs of the *Arabidopsis AGAMOUS (AG)* protein. A neighbor-joining tree illustrates the phylogenetic relationship between homologs of the *Arabidopsis AG* protein. Nodes with less than 70% bootstrap support are indicated with grey triangles. Except for *Zea mays*, which was included as outgroup species, the taxa within the tree are either members of the *Brassicaceae* subfamily or the *Asteridae* class. Residues behind taxon names correspond to the AIP sequence segment and an asterisk indicates that only the inserted residue(s) were found. No insertions were found in *Arabidopsis* and in *P. hybrida*. The distinct insertions found in *R. raphanistrum* might be the result of different allelic variants or sequencing errors. Note that the sequences from the taxa within the *Asteridae* are homologs of the *FARINELLI (FAR)* gene in *Antirrhinum majus.* The full names of the species used in this tree are provided in the Material and Methods section. The alignment corresponding to this tree is provided in Figure S5.

Third, *Arabidopsis AGAMOUS (AG), SHATTERPROOF1 (SHP1),* and *SHATTERPROOF2 (SHP2)* are paralogs that originated from a common ancestral gene through two independent duplication events. The first gave rise to the *AG* and *SHP* ancestor lineages and the second led to the *SHP1* and *SHP2* paralogs [49]. Although no AS events were detected for the *Arabidopsis AG* locus, conserved AIPs were identified in *AG* homologs from seven species (Figure 3). The conserved AIP within the Asteraceae involved a single Q-residue whereas the conserved AIP within the Brassicaceae involved three amino acid residues. The intron position corresponding to the two-residue AIP in the *SHP2* gene is orthologous to the intron position corresponding to the conserved AIP in *AG* in non-*Arabidopsis* species. Both the *SHP2* and *AG* loci encode an interaction motif that spans this intron position. Recently, it was shown that insertion of a single amino-acid into the predicted motif in the AG protein eliminates its ability to induce female organ development in the first whorl [50]. In addition, it was shown that similar phenotypic differences are induced by the presence or absence of a single glutamine residue within a sequence region in the FARINELLI (FAR) protein of *Antirrhinum majus* that is homologous to the motif in the AG protein. The motif in the AG protein is almost exactly conserved in the homologous sequences from the species with the conserved AIPs (Figure S6).

**Figure 4:**
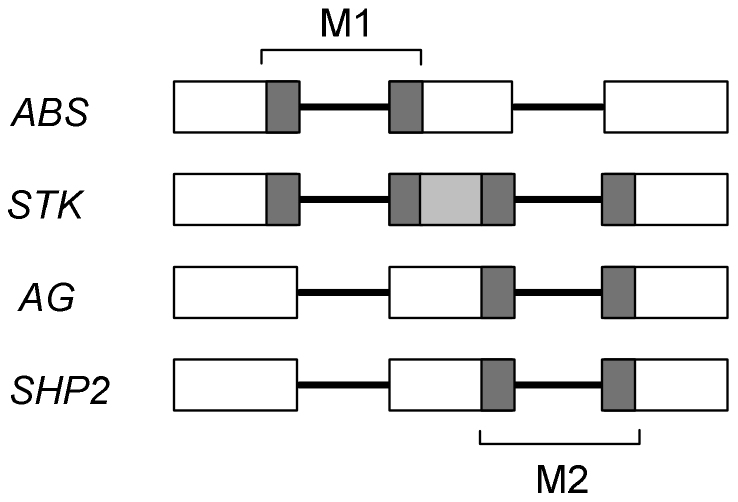
Homologous interaction motifs overlapping with AIPs. Conserved intron positions of the *Arabidopsis ABS*, *STK*, *AG* and *SHP2* genes are represented by horizontal lines and their flanking exons by rectangles. Grey colored regions correspond to predicted interaction motifs that overlap with AIPs. The light grey motif only overlaps the AIP in *STK*. **M1**: Homologous interaction motifs that overlap with AIPs in *ABS* and *STK*. **M2**: Homologous interaction motifs that overlap with AIPs in *STK*, *AG*, and *SHP-2*.

The alternatively spliced exon of the *Arabidopsis STK* gene encodes three consecutive interaction motifs, two of which span the introns flanking the exon. The motif spanning the 5′-intron is homologous to the motif that is affected by the AS event of the *ABS* locus. On the other hand, the motif that spans the 3′-intron is homologous to the motif that overlaps with the AIP of *SHP2*. The latter motif is also homologous to the conserved motifs that overlap with the AIPs in the non-*Arabidopsis* species (Figure 4).

### AS in the evolution of the FLOWERING LOCUS C (FLC)-clade

**Figure 5:**
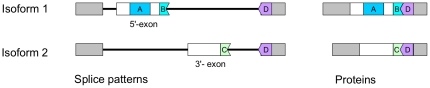
Interaction motif architecture of *MADS AFFECTING FLOWERING1 (MAF1)* isoforms. Two *MADS AFFECTING FLOWERING1* (*MAF1*) isoforms differ as the result of the selective inclusion of either one of the two mutually exclusive exons (named 5′- and 3′-exon). Only the 5′-exon of the mutually exclusive pair that is included in isoform 1 contains motif **A**. Both the 5′- and 3′-exons of the mutually exclusive pair have residues at their 3′-boundary that can form interaction motifs (**BD** or **CD**) together with the first residues of the downstream constitutively spliced exon. Introns are indicated by horizontal lines.

As mentioned above, the mutually exclusive exon event of the *MAF1* locus results in the production of protein isoforms with different interaction motif architectures in the I-domain (Figure 5). The proteins encoded by the transcripts containing the 5′-exon have one interaction motif more than those encoded by transcripts that include the 3′-exon. Both exons encode for amino acid residues at their 3′-site that combine with the first residues of the downstream constitutively spliced exon to form interaction motifs. Hence, the mutually exclusive exon event provides a “switch” between two interaction motifs. The 5′-exon could be detected in transcripts from all other *FLC*-like clade members (Figure S7). A BlastN search [51] with the 3′-exon (cryptic exon) against the genome of *Arabidopsis* revealed the presence of highly similar sequences within the second introns of only the *MAF2* and *MAF3* loci (Figure S8A). In fact, the nucleotide identity is high over the entire length of the alignment between the introns.

The results obtained using the BlastN search were confirmed by additional Smith and Waterman alignments [52] between the 3′-cryptic exon of *FLM* and the second intron (target introns in Figure S7) of each of the other FLC-clade members (data not shown).

The intronic region of the *MAF2* locus that is homologous to the cryptic exon of *MAF1* can be translated fully in the same frame as the *MAF1* exon (Figure S8B). However, the nearest acceptor site is located 2 nt upstream of the 5′-site of the region corresponding to the cryptic exon. Usage of this acceptor site would result in a frame shift followed by a premature termination codon. Inspection of the translated intronic region of *MAF3* revealed the presence of an in-frame termination codon. Hence, this region cannot be fully incorporated into a protein sequence.

## Discussion

In this study we performed a systematic analysis of AS within the MIKC-type MADS-box transcription factor family in *Arabidopsis*. Our study was focused on the functional impact of AS on different aspects of the formation of protein complexes. We restricted our analysis to transcripts which are likely to be translated, excluding those AS transcripts that are candidate to be degraded by the NMD-pathway. Nevertheless, we cannot exclude potential functions of these transcripts, which would only further add to the importance of AS in the MADS domain family.

Although AS events were not significantly overrepresented in any of the DNA regions encoding conserved protein domains, intron clusters within the K-box and C-terminal domain encoding sequences were twice less frequently involved in AS events than intron clusters located in the I-domain encoding DNA sequences. Although the I-domain is not a predicted PFAM domain, it has a known function in determining dimerization specificity [16], [24].

AS events that overlapped with interaction motifs within the I-domain encoding DNA sequences were shown to potentially affect dimer interaction specificities. Indeed, of these three loci, the *SVP* locus was predicted to have isoforms with different interactions specificities and this was also confirmed by yeast two-hybrid analyses [24]. Ectopic expression demonstrated clear differences in function between the two *SVP* AS isoforms; overexpression of *SVP1* resulted in a late flowering phenotype and floral abnormalities, whereas overexpression of *SVP3* did not. The observed differences in effects on flowering-time are in line with the expectations based on protein-protein interactions: only SVP1 is able to interact with the strong repressor of flowering FLOWERING LOCUS C (FLC) [24] and previously it was shown that the SVP and FLC function in floral repression is mutually dependent [53]. The same holds for the differences in observed floral defects, which probably are at least partially caused by direct interactions between SVP1 and the ABC-class MADS domain proteins, a capacity that SVP3 is lacking [24]. Altogether, these observations show that a small change in the sequence of a MADS-box gene transcript due to an AS event can have strong consequences for the interaction capacity and functioning of the encoded protein. Nevertheless, the *SVP3* transcript is lowly expressed in comparison to the fully spliced *SVP1* transcript and the elucidation of its true biological function requires further experimental investigations.

It has been demonstrated that AS can be associated with fast evolving regions of proteins [54]. The elevated rate of evolution of the less well conserved I-domain [55] is not only evident from the amino acid substitutions in this region but also from the repeated changes that have occurred to its intron structure [19]. The increased rates of AS in this region represent a further mechanism to diversification and the overall high variability of the I-domain may reflect its role as a determinant of dimer interaction specificity.

The potential effect of an AS event in the K-box depends on the location of the event within this domain. In accordance with previously published data [21] we hypothesize that the interaction motif that is introduced through an intron retention event located in the sequence encoding for the N-terminal region of the MAF1 K-box domain has a potential effect on dimer formation. AS events that affected the C-terminal region of the K-box were considered to influence multimer formation, because this region was shown to be important for higher-order complex formation [18]. Based on the rather similar positions of their AS events, we hypothesize that the variants encoded by the *STK-* and *SHP2-*loci form different higher-order complexes, similar to what has been shown experimentally for the ABS isoforms [18].

All AIPs that showed conservation across species were located at the boundary of, or within the DNA sequence encoding the K-box region and can roughly be divided into two groups. The first group consists of the AIPs from the *Arabidopsis SEP3*-, *MAF2* and *MAF3*-loci that were located near the sequence encoding the N-terminal boundary of the K-box domain. Although none of these AIPs involved residues corresponding to interaction motifs, the positions of these AIPs suggest that they might influence dimer formation [16]. The second group contains conserved AIPs that overlapped with DNA sequences encoding predicted interaction motifs located near the C-terminal region of the K-box domain. The motifs involved in these AIPs were all encoded in orthologous exons. The motifs spanning 5′- and 3′-introns of the skipped exon of the *STK* gene are homologous to the motifs that overlap with the AIPs of *ABS* and both *SHP2* and *AG* (in the non*-Arabidopsis* species), respectively.

Previous comparative analyses in plants have shown that AS events are generally not well conserved [46], [47], [48]. Conservation is even less pronounced when not only the location but also the effect of the event on the protein sequence is taken into account [4]. It is therefore remarkable that conserved AIPs were here found for six members of the same gene family. In contrast to these previous large-scale studies, our present conservation analysis was not limited to a few species with large fractions of their genome sequenced. Instead, we searched for conserved AIPs in a large collection of EST/cDNA sequences from a wide variety of species. Most of the conserved AIPs in this study were found between closely related species and are congruent with the phylogenetic relationships of the surrounding sequences. While this is not unexpected, it is less clear whether those AIPs that span entire exons in *Arabidopsis*, as found for the STK and ABS homologs, are true cases of conservation. These AIP types might be easier to detect at larger distances because exons are in general much better conserved than intronic sequences. As these AIPs clearly contain sequences that resemble the interaction motifs detected in *Arabidopsis*, the effect of the AIP might also resemble the potential effect in *Arabidopsis*.

The importance of considering the effect of AS on conserved interaction motifs is highlighted by a recent study [50]. It was shown that observed phenotypic differences could be explained by the inclusion or exclusion of a single amino acid into a conserved sequence region from the *Arabidopsis* AG and the *Antirrhinum* FAR proteins. This phenotypic difference is caused at the molecular level by altered interaction specificities resulting from the presence or absence of the single amino-acid. Interestingly, the single amino acid insertion into FAR that is the crucial difference responsible for functional diversification between this protein and its paralog *PLENA* (PLE) is identical to our observed differences between AS variants of AG homologs. From an evolutionary perspective, FAR and PLE could represent two ancient AS isoforms that have been partitioned over separate genes. Although a number of cases of such externalization [56] events have been described in literature [56], [57], [58], the extent to which this process has contributed to the protein diversity in plants is not known.

*MAF1* is a member of the *FLC*-like clade in *Arabidopsis* which consists of genes that are subject to extensive alternative splicing and genomic rearrangements [31], [39], [40], [59]. An interesting aspect of the *MAF1* locus is the complexity of the rearrangement of motif content that results from a mutually exclusive exon event. While the 5-exon of the mutually exclusive exon pair could be detected in transcripts of other *FLC*-like genes, remnants of the 3′-cryptic exon were only detected in the closely related *MAF2-* and *MAF3* loci. As the *MAF1–3* loci form a monophyletic group within the *FLC*-like clade (e.g. Figure 6 in [15]) it is likely that the cryptic exon originated recently within the ancestor of this group. Alternative splicing has previously been associated with the emergence of new exons [60]. In accordance with the view that AS provides genes with “internal paralogs” [61], the recent exon provides the material for these *MAF* proteins to “experiment” with new dimer interactions without the irreversible loss of existing interactions. The *MAF2-* and *MAF3* loci are not likely to express the region corresponding to the cryptic exon at the protein level. Such rapid divergence of AS patterns between recent paralogs has previously been demonstrated to be a common phenomenon [62], [63].

Naturally occurring mutations in splice sites that affect splicing patterns have been identified in *FLOWERING LOCUS C (FLC)* related genes in different species. An *FLC* gene has been identified in the *Arabidopsis* Bur-0 accession that produces a transcript encoding a protein with a modified C-domain (both insertion and truncation) due to a mutation affecting the acceptor site of the sixth intron [64]. In that same study it was reported that the *FLC* gene of the Van-0 accession has a nonsense mutation in the sixth exon. The gene produces two transcripts that differ by the presence of the sixth exon. Inclusion and exclusion of the sixth exon lead to a protein with a truncated C-domain or large deletion in the C-domain, respectively. Although the mutation in the Van-0 accession does not directly affect a splice site, it can affect a potential *cis*-element such as a splicing enhancer element that might be needed for correct recognition of this exon which is very short compared to the average *Arabidopsis* exon (42 nt. versus 217 nt on average [65]). Natural variation affecting the donor site of the sixth intron of the *FLC* gene in *B. rapa* has been reported [66]. Individuals carrying this mutation produce two transcripts in which either an alternative donor site within intron 6 is used or similarly as in the Van-0 *Arabidopsis* accession, the sixth exon is skipped entirely. It is however not known whether the early flowering phenotypes associated with these reported *Arabidopsis* and *B. rapa FLC* genes are the result of reduced multimer formation capabilities of FLC or reduced transcription activation capabilities.

Another naturally occurring splice site mutation in *Capsella bursa-pastoris* results in the skipping of the fifth *FLC A* exon and is associated with early flowering [67]. This exon corresponds to the fourth exon of the *Arabidopsis ABS* gene which encodes the earlier mentioned motif involved in higher order interaction specificities. We therefore hypothesize that the early flowering phenotype associated with this exon skipping event is the result of altered multimer formation of *FLC A*.

In addition to these cases in which *cis* splice signals were genetically altered, cases have been found in which the relative abundance of the splice variants from *FLC* related genes is regulated in a manner dependent on temperature [31], [68], [69] or developmental stage [70]. These findings suggest a “conserved” usage of the alternative splicing process as mechanism for regulating flowering-time. However, the details of flowering-time regulation can differ between species even if the AS events in homologous genes appear to be conserved. For instance, the temperature-dependent skipping of the fifth exon in the *Beta vulgaris FLC like* 1 gene results in transcripts encoding proteins with stronger repressor capabilities than those encoded by transcripts that include the exon [69]. The fifth exon that is skipped in *C. bursa-pastoris FLC A* transcripts is homologous to the skipped exon in *B. vulgaris* (data not shown). However, *C. bursa-pastoris* transcripts that contain the exon are stronger repressors and those transcripts that lack the exon are associated with early flowering phenotypes [67]. Although the proposed effects of the AS products are opposite, these findings provide strong evidence that AS in the *FLC*-clade genes plays an important role in flowering-time regulation. Based on our results and predictions and observations done previously (reviewed in [71]) it is tempting to speculate that AS in general, and in particular for MADS-box transcription factors, is an important molecular mechanism underlying flowering-time regulation.

Considered at the level of the gene family, the impact of AS on the MIKC subgroup does not appear to dramatically differ from previously established genome-wide patterns. For instance, the dynamic nature of the process is emphasized by the association between AS and fast evolving protein regions and the rapid divergence of AS in recent paralogs, which we find here but is also known on a genome-wide scale. However, usage of available detailed annotation of specific functions to various sequence regions enables the identification of AS events that have an impact on function but that remain hidden behind the globally observed patterns. Such sequence annotations are more specific than for example PFAM domains, and although such knowledge is often not found in databases but somewhat hidden in the literature, for many protein families experimental and predicted sequence regions are known that impact aspects of protein functions. Hence, the results of our study are encouraging for future analysis of AS within other gene families following a similar approach.

To conclude, a large fraction of the AS events in genes encoding MADS domain proteins that are likely to be manifested at the proteome level are predicted to be functional according to our criteria. For many of these cases, hypotheses could be formulated about aspects of protein complex formation that are affected by the AS events. Using additional experimental data (qRT-PCR, overexpression studies) as well as additional bioinformatics analysis (translatome data) we present supporting evidence for the potential biological relevance of the predicted functional AS events. In addition to demonstrating the functional impact of AS on extant MIKC proteins, we also illustrated how the process can potentially introduce new interactions into the network. The analysis we present here provides a starting point for further experimental determination of the precise physiological roles of AS events in plant MADS-box genes.

## Materials and Methods

### Initial data

The identifiers corresponding to the *Arabidopsis* MADS-box genes were extracted from the “gene_families_sep_29_09_update.txt” file, which was downloaded from the TAIR website (www.Arabidopsis.org). The genome and predicted proteome of *Arabidopsis thaliana* version TAIR 10.0 were used in this study. cDNA and EST sequences of *Arabidopsis* were downloaded from the PlantGDB webpage [72]. Gene models and AS events were predicted using our previously described method [4]. Only those TAIR loci were considered that had at least one annotated protein with an open reading frame (ORF) that was fully supported by cDNA/EST evidence. Transcripts containing premature termination codons (PTCs) located more than 55 nt upstream of the last exon/intron boundary were considered to be NMD targets [43].

### Domain annotation

The PFAM [73] domain architectures of the MADS-proteins were determined using InterProScan [74]. Only those MIKC proteins encoding both the MADS- and K-domains were considered further. A multiple sequence alignment of MADS proteins with clear MIKC structures was built using clustalX2 [75] in order to designate the boundaries in accordance with a previously published structural annotation (Figure 1 in [76]).

### Interaction motifs and protein-protein interaction

Putative interaction motifs and protein-protein interactions were predicted using our recently developed IMSS predictor [24]. In brief, using protein sequences and yeast two-hybrid interaction data, pairs of short sequence motifs are found that occur more often in pairs of interacting proteins compared to pairs of non-interacting proteins. In this motif search, only *Arabidopsis* sequences were used, but the obtained interactions motifs were subsequently found to display reasonably strong conservation. Additional validation was obtained using various bioinformatics analyses of those motifs, and most importantly using experimental mutagenesis on motif sites to change interaction specificities of various MADS-domain proteins. The predictions were only done for protein isoforms of loci that were represented in the previously published interaction map of *Arabidopsis* MADS-box genes [17]. Visualization of the SVP1 and SVP3 interaction network was done using Cytoscape version 2.8.1 [77].

### Homology searches

The “est_others” file was downloaded from the NCBI blast database ftp site (ftp://ftp.ncbi.nih.gov/blast/db/FASTA/) and translated using the getorf program from the EMBOSS package [78] version 5.0. BlastP [51] (complexity filter off; max e-value: 1e^−10^) searches were performed with the translated sequences against the *Arabidopsis* proteome. Only the best matches involving an *Arabidopsis* MIKC protein were kept, given that the corresponding alignment had an identity of at least 40% and included at least 60% of the residues of the *Arabidopsis* protein.

### Identification of AS-induced polymorphism (AIP)

CAP3 [79] (-p 95–o 100) was used for clustering ESTs from a single species that had a protein from the same *Arabidopsis* locus as their best blast hit. QualitySNP [80] was used for dividing these initial clusters into groups of sequences that were likely to represent the same allelic gene variant. The sequences in these groups were assembled into final transcript contigs using CAP3.

The identification of AIPs relied on the assumption that intron positions within the coding region of genes are generally well conserved. This assumption is based on a previous study in which it was demonstrated that around 94% of introns within DNA stretches encoding conserved protein regions are shared between *Arabidopsis* and rice [81]. The positions of putative introns in the subject species were mapped by constructing Needleman-Wunsch [82] alignments between the subject sequences and their corresponding *Arabidopsis* homologs with known gene structures.

AIPs were identified as indels that coincided with putative intron positions on global alignments between proteins that were predicted to be isoforms of the same gene. It was required that ten alignment positions immediately flanking the polymorphic region contained at least four identical residue pairs and at most one gapped position. It was furthermore required that one of the two isoform sequences could be aligned to the *Arabidopsis* homolog without any gaps around the intron position. As a result of this requirement, AIPs could be represented by amino acid sequences. AIPs were considered to be conserved when the global alignment between corresponding sequences were at least 40% similar.

### AS isoform expression studies

Expression of the putative AS events was analyzed by qRT-PCR. Based on known expression patterns of the concerning genes, either cDNA from leaves or carpels was used. For leaf material the full-grown first leaves from five individual plants were taken and carpels were isolated from flowers just prior to opening. RNA was isolated by lithium chloride-phenol-chloroform extraction [83] followed by DNase (Invitrogen) treatment. One microgram RNA was used to perform cDNA synthesis using M-MuLV Reverse Transcriptase (Promega). The cDNA made this way was diluted four times and used for quantitative Real-Time PCR (qRT-PCR) using the SYBR green mix from BioRad. At5g08290, which was determined as “superior reference gene” [84], was used as reference for the analyses. As control for DNA contamination a minus RT reaction was performed. As second control we performed a “non-template” reaction. All reactions were done in duplo. The oligonuceotides used in the transcript analyses are given in Table S3. No direct quantitative comparison of expression levels of the individual isoforms can be made based on the qRT-PCR results, because of the usage of the different isoform specific primer-sets. Though, quality of the designed primers was analyzed by performing a primer efficiency test. Furthermore, specificity of oligonucleotides was determined by sequencing (DETT sequencing, Amersham) of the amplified fragments.

### Analysis of translatome data

As additional validation for the relevance of the selected AS events, the short sequence reads from the study of Jiao and Meyerowitz [44] were mapped against the *Arabidopsis* genome using the program TopHat version 1.3.0 [85]. All the bam files created by TopHat were merged into a single file using samtools version 0.1.16 [86]. The total number of reads that supports the individual variants associated with each of the predicted functional AS events were determined using the MISO package [87] as of 12 July 2011.

### *SVP* overexpression studies

Entry vectors containing the full length coding sequences of *SVP1* (At2g22540.1) and *SVP3* (At2g22540.2) [24] were recombined via a Gateway LR reaction (Invitrogen, Carlsbad) with a binary destination vector containing a *CaMV35S* promoter for the purpose of ectopic expression. Generated expression vectors CZN094 (*pCaMV35S::SVP1:NosT*) and CZN756 (*pCaMV35S::SVP3:NosT*) were transformed into *Agrobacterium tumefaciens* strain C58C1/PMP90 followed by transformation of *Arabidopsis thaliana* Col0 wild type plants using the standard floral dip method [88]. Primary transformants were selected based on selective germination and analysis of expression levels of the ectopically expressed *SVP1* and *SVP3* transgenes by qRT-PCR (for oligonucleotide sequences see Table S3). Possible effects on flowering-time were analysed by growing a population of progeny plants (n = 25) from three selected independent primary transformants of CZN094 and CZN756, respectively. As a control Col0 wild type plants were analysed (n = 15). Plants were grown under short day conditions (8 hours light, 16 hours dark; 21°C). For each individual plant the number of days from sowing until bolting was scored, as well as the number of rosette leaves at the moment of bolting. Statistical significance was determined using Student's *t*-test (p<0.01).

### Phylogenetic analysis of *Arabidopsis* AGAMOUS homologs

A multiple protein sequence alignment of identified *Arabidopsis* AG homologs was constructed using ClustalX2 and trimmed at the column corresponding to the first residue of the Arabidopsis protein. ClustalX2 was also used for creating a consensus neighbor-joining tree [89] from 1000 bootstrap replicates generated from the trimmed alignment. Gapped positions were excluded and a correction was applied for multiple substitutions during the tree construction procedure. The consensus tree topology was edited using the tree explorer from the MEGA package [90] version 4. The following specific varieties or subspecies as annotated in the NCBI taxonomy database [91] are represented in the phylogenetic tree: *Raphanus raphanistrum subsp*. *raphanistrum*, *Brassica rapa subsp*. *pekinensis*, *Raphanus sativus var*. *oleiformis*, *Petunia axillaris subsp*. *axillaris* and *Pentunia x hybrida*.

## Supporting Information

Figure S1 **Conserved AIPs in homologs of the Arabidopis** ***SEPALLATA3 (SEP3)*** **protein.** The intron position corresponding to the AIP of the Arabidopsis *SEP3* isoforms is indicated by the black triangle. *A.thaliana*-1 and *A.thaliana*-2 correspond to *SEP3.1* and *SEP3.2*, respectively.
(DOC)

Figure S2 **Conserved AIPs in Brassica homologs of the Arabidopsis** ***MADS AFFECTING FLOWERING 2 and −3 (MAF2 and MAF3)*** **proteins.** The conserved intron position corresponding to both the AIPs of the Arabidopsis *MAF2-* and *MAF3* isoforms is indicated by the black triangle. B.rapa.p corresponds to *Brassica rapa subsp. pekinensis.* MAF3-short and MAF3-long correspond to *MAF3.2* and *MAF3.1*, respectively. MAF2-long corresponds to *MAF2.2*.
(DOC)

Figure S3 **Conserved AIP for the Arabidopsis** ***SEEDSTICK (STK)*** **protein.** The introns flanking the skipped exon corresponding to the AIP of the Arabidopsis *STK* isoforms are indicated by black triangles. The *A.thaliana*-short and *A.thaliana*-long isoforms correspond to *STK.1* and *STK.2*, respectively.
(DOC)

Figure S4 **Conserved AIPs between homologs of the Arabidopsis** ***B-sister (ABS)*** **protein.** The location of the intron corresponding to the AIP in Arabidopsis is indicated by the grey triangle. The conserved AIP between *R. communis* and *G. hirsutum* corresponds to an entire exon in Arabidopsis. The position of the intron flanking the 3′ - side of the Arabidopsis exon is indicated by the black triangle. The *A.thaliana*-short and *A.thaliana*-long isoforms correspond to *ABS.2* and *ABS.1*, respectively.
(DOC)

Figure S5 **Amino-acid Alignment of Arabidopsis AGAMOUS homologs.** The alignment contains translated EST assemblies for the species represented in the phylogentic three in figure 3 of the main text. The alignment has been trimmed to the N-terminal residue of the *A.thaliana* protein. The EST assembly method is provided in the material and methods section of the main text.
(DOC)

Figure S6 **Conservation of a predicted interaction motif in Arabidopsis AGAMOUS homologs.**
(DOC)

Figure S7 **Alignment of Arabidopsis FLC-clade members.** For each sequence in the alignment both its short and AtNumber (TAIR identifier) are provided. The positions of the introns with the coding region correspond to the aligned proteins are indicated using the characters 0, 1 or 2 which correspond to the phase of the intron. The aligned-positions containing the protein sequences encoded by the exons that are homologous to the 5′- exon of the mutually exclusive exon pair in *MAF1* (see main text) are underlined. The target introns are those introns that have been analyzed for the presence of sequences which are homologous to the 3′-exon of the mutually exclusive exon pair of *MAF1* (see main text).
(DOC)

Figure S8 **Conservation of the** ***MADS AFFECTING FLOWERING1 (MAF1)*** **cryptic exon sequence.** **A.** Multiple sequence alignment of the second intron from the Arabidopsis *MAF1–3* genes. The conserved region corresponding to the 3′-cryptic exon from the mutually exclusive exon pair in the *MAF1* gene is highlighted by the shaded box. **B.** Multiple sequence alignment of the translated intronic regions from the *MAF2*- and *MAF3* gene and homologous 3′- cryptic exon of *MAF1*.
(DOC)

Table S1 **MADS-box gene data.** For each TAIR 10 locus that is annotated as a MADS-box gene it is indicated whether at least one predicted protein has: (*i*) an open reading frame fully supported by transcript data; (*ii*) experimental dimer interaction data, and (*iii*) a clear MIKC structure. It is further indicated whether a gene is alternatively (A) or constitutively spliced (C), whether a gene produces transcripts that are NMD targets and/or transcripts that are predicted to be translated into proteins. The number of inferred AS events and the annotated protein names are also given.
(DOC)

Table S2 **Effect of AS on predicted interaction motifs.** The residues from each motif that overlap with AIPs are red. Residues that are inserted into motifs are surrounded by square brackets. Multiple overlapping interaction motifs that are affected by the same AIP are stacked. Multiple non-overlapping motifs that are affected by the same AS event are numbered.
(DOC)

Table S3 **Sequences of oligonucleotides used in this study.** The first listed oligonucleotide is always the forward primer and the second one the reverse.
(DOC)
