# Supplementary material for: Predicting the Impact of Alternative Splicing on Plant MADS Domain Protein Function
Source: PLoS One. 2012 Jan 25;7(1):e30524. doi: 10.1371/journal.pone.0030524 (PMC3266260; doi:10.1371/journal.pone.0030524)
Supplement: Figure S1 — Conserved AIPs in homologs of the Arabidopis SEPALLATA3 (SEP3) protein. The intron position corresponding to the AIP of the Arabidopsis SEP3 isoforms is indicated by the black triangle. A.thaliana-1 and A.thaliana-2 correspond to SEP3.1 and SEP3.2, respectively. (DOC) [file pone.0030524.s001.doc]

**Figure S1. Conserved AIPs in homologs of the Arabidopis *SEPALLATA3 (SEP3)* protein.** The intron position corresponding to the AIP of the Arabidopsis *SEP3* isoforms is indicated by the black triangle. *A.thaliana*-1 and *A.thaliana*-2 correspond to *SEP3.1* and *SEP3.2*, respectively.
