# Supplementary material for: Predicting the Impact of Alternative Splicing on Plant MADS Domain Protein Function
Source: PLoS One. 2012 Jan 25;7(1):e30524. doi: 10.1371/journal.pone.0030524 (PMC3266260; doi:10.1371/journal.pone.0030524)
Supplement: Figure S2 — Conserved AIPs in Brassica homologs of the Arabidopsis MADS AFFECTING FLOWERING 2 and −3 (MAF2 and MAF3) proteins. The conserved intron position corresponding to both the AIPs of the Arabidopsis MAF2- and MAF3 isoforms is indicated by the black triangle. B.rapa.p corresponds to Brassica rapa subsp. pekinensis. MAF3-short and MAF3-long correspond to MAF3.2 and MAF3.1, respectively. MAF2-long corresponds to MAF2.2. (DOC) [file pone.0030524.s002.doc]

**Figure S2. Conserved AIPs in Brassica homologs of the Arabidopsis *MADS AFFECTING FLOWERING 2 and -3 (MAF2 and MAF3)* proteins.** The conserved intron position corresponding to both the AIPs of the Arabidopsis *MAF2-* and *MAF3* isoforms is indicated by the black triangle. B.rapa.p corresponds to *Brassica rapa subsp. pekinensis.* MAF3-short and MAF3-long correspond to *MAF3.2* and *MAF3.1*, respectively. MAF2-long corresponds to *MAF2.2*.
