# Supplementary material for: Predicting the Impact of Alternative Splicing on Plant MADS Domain Protein Function
Source: PLoS One. 2012 Jan 25;7(1):e30524. doi: 10.1371/journal.pone.0030524 (PMC3266260; doi:10.1371/journal.pone.0030524)
Supplement: Figure S3 — Conserved AIP for the Arabidopsis SEEDSTICK (STK) protein. The introns flanking the skipped exon corresponding to the AIP of the Arabidopsis STK isoforms are indicated by black triangles. The A.thaliana-short and A.thaliana-long isoforms correspond to STK.1 and STK.2, respectively. (DOC) [file pone.0030524.s003.doc]

**Figure S3. Conserved AIP for the Arabidopsis *SEEDSTICK (STK)* protein***.* The introns flanking the skipped exon corresponding to the AIP of the Arabidopsis *STK* isoforms are indicated by black triangles. The *A.thaliana*-short and *A.thaliana*-long isoforms correspond to *STK.1* and *STK.2*, respectively.
