# Supplementary material for: Predicting the Impact of Alternative Splicing on Plant MADS Domain Protein Function
Source: PLoS One. 2012 Jan 25;7(1):e30524. doi: 10.1371/journal.pone.0030524 (PMC3266260; doi:10.1371/journal.pone.0030524)
Supplement: Figure S4 — Conserved AIPs between homologs of the Arabidopsis B-sister (ABS) protein. The location of the intron corresponding to the AIP in Arabidopsis is indicated by the grey triangle. The conserved AIP between R. communis and G. hirsutum corresponds to an entire exon in Arabidopsis. The position of the intron flanking the 3′ - side of the Arabidopsis exon is indicated by the black triangle. The A.thaliana-short and A.thaliana-long isoforms correspond to ABS.2 and ABS.1, respectively. (DOC) [file pone.0030524.s004.doc]

**Figure S4. Conserved AIPs between homologs of the Arabidopsis *B-sister (ABS)* protein.** The location of the intron corresponding to the AIP in Arabidopsis is indicated by the grey triangle. The conserved AIP between *R. communis* and *G. hirsutum* corresponds to an entire exon in Arabidopsis. The position of the intron flanking the 3’ - side of the Arabidopsis exon is indicated by the black triangle. The *A.thaliana*-short and *A.thaliana*-long isoforms correspond to *ABS.2* and *ABS.1*, respectively.
