# Supplementary material for: Predicting the Impact of Alternative Splicing on Plant MADS Domain Protein Function
Source: PLoS One. 2012 Jan 25;7(1):e30524. doi: 10.1371/journal.pone.0030524 (PMC3266260; doi:10.1371/journal.pone.0030524)
Supplement: Figure S5 — Amino-acid Alignment of Arabidopsis AGAMOUS homologs. The alignment contains translated EST assemblies for the species represented in the phylogentic three in figure 3 of the main text. The alignment has been trimmed to the N-terminal residue of the A.thaliana protein. The EST assembly method is provided in the material and methods section of the main text. (DOC) [file pone.0030524.s005.doc]

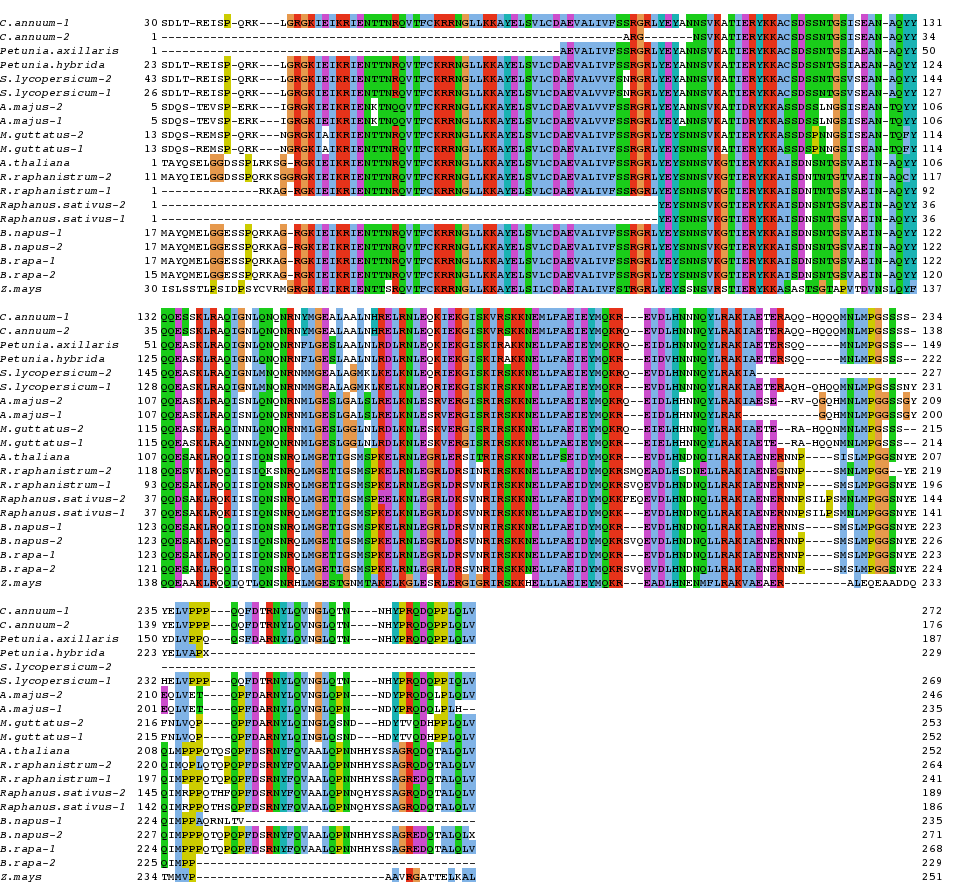


**Figure S5. Amino-acid Alignment of Arabidopsis AGAMOUS homologs.** The alignment contains translated EST assemblies for the species represented in the phylogentic three in figure 3 of the main text. The alignment has been trimmed to the N-terminal residue of the *A.thaliana* protein. The EST assembly method is provided in the material and methods section of the main text.
