# Supplementary material for: Predicting the Impact of Alternative Splicing on Plant MADS Domain Protein Function
Source: PLoS One. 2012 Jan 25;7(1):e30524. doi: 10.1371/journal.pone.0030524 (PMC3266260; doi:10.1371/journal.pone.0030524)
Supplement: Figure S6 — Conservation of a predicted interaction motif in Arabidopsis AGAMOUS homologs. (DOC) [file pone.0030524.s006.doc]

**Figure S6. Conservation of a predicted interaction motif in Arabidopsis AGAMOUS homologs.**
