# Supplementary material for: Predicting the Impact of Alternative Splicing on Plant MADS Domain Protein Function
Source: PLoS One. 2012 Jan 25;7(1):e30524. doi: 10.1371/journal.pone.0030524 (PMC3266260; doi:10.1371/journal.pone.0030524)
Supplement: Figure S7 — Alignment of Arabidopsis FLC-clade members. For each sequence in the alignment both its short and AtNumber (TAIR identifier) are provided. The positions of the introns with the coding region correspond to the aligned proteins are indicated using the characters 0, 1 or 2 which correspond to the phase of the intron. The aligned-positions containing the protein sequences encoded by the exons that are homologous to the 5′- exon of the mutually exclusive exon pair in MAF1 (see main text) are underlined. The target introns are those introns that have been analyzed for the presence of sequences which are homologous to the 3′-exon of the mutually exclusive exon pair of MAF1 (see main text). (DOC) [file pone.0030524.s007.doc]

*FLC-AT5G10140.1* -------MGRKKLEIKRIENKSSRQVTFSKRRNGLIEKARQLSVLCDASVALLVVSASGKLYSFSSGD2NLVKILDRYGK

*MAF1-AT1G77080.4* -------MGRRKIEIKRIENKSSRQVTFSKRRNGLIDKARQLSILCESSVAVVVVSASGKLYDSSSGD2DISKIIDRYEI

*MAF2-AT5G65050.1* -------MGRKKVEIKRIENKSSRQVTFSKRRNGLIEKARQLSILCESSIAVLVVSGSGKLYKSASGD2NMSKIIDRYEI

*MAF3-AT5G65060.1* -------MGRRKVEIKRIENKSSRQVTFSKRRKGLIEKARQLSILCESSIAVVAVSGSGKLYDSASGD2NMSKIIDRYEI

*MAF4-AT5G65070.1* -------MGRRKVEIKRIENKSSRQVTFCKRRNGLMEKARQLSILCESSVALIIISATGRLYSFSSGD2SMAKILSRYEL

*MAF5-AT5G65080.1* MCRKSEAMGRRRVEIKRIENKSSRQVTFCKRRNGLMEKARQLSILCGSSVALFIVSSTGKLYNSSSGD2SMAKIISRFKI

*FLC-AT5G10140.1* QHADDLKAL0DHQSKALNYGSHYELLELVD2SKLVGSNVKNVSIDALVQLEEHLETALSVTRAKK0TELMLKLVENLKEK

*MAF1-AT1G77080.4* QHADELRAL0DLEEKIQNYLPHKELLETVQ2SKLEEPNVDNVSVDSLISLEEQLETALSVSRARK0AELMMEYIESLKEK

*MAF2-AT5G65050.1* HHADELEAL0DLAEKTRNYLPLKELLEIVQ2SKLEESNVDNASVDTLISLEEQLETALSVTRARK0TELMMGEVKSLQKT

*MAF3-AT5G65060.1* HHADELKAL0DLAEKIRNYLPHKELLEIVQ2SKLEESNVDNVSVDSLISMEEQLETALSVIRAKK0TELMMEDMKSLQER

*MAF4-AT5G65070.1* EQADDLKTL0DLEEKTLNYLSHKELLETIQ2CKIEEAKSDNVSIDCLKSLEEQLKTALSVTRARK0TELMMELVKTHQEK

*MAF5-AT5G65080.1* QQADDPETL0DLEDKTQDYLSHKELLEIVQ2RKIEEAKGDNVSIESLISMEEQLKSALSVIRARK0TELLMELVKNLQDK

↑

**Target introns**

*FLC-AT5G10140.1* 0EKMLKEENQVLASQ---0MEN-NHHVGAEAEME-MSPAGQISDNLPVTLPLLN

*MAF1-AT1G77080.4* 0EKLLREENQVLASQ---0MGK-NTLLATDDERG-MFPGSSSGNKIPETLPLLN

*MAF2-AT5G65050.1* 0-----------------_VGK-KTFLVIEGDRG-MSWENGSGNKVRETLPLLK

*MAF3-AT5G65060.1* 0EKLLIEENQILASQ---0VGK-KTFLVIEGDRG-MSRENGSGNKVPETLSLLK

*MAF4-AT5G65070.1* 0EKLLREENQSLTNQLIK0MGKMKKSVEAEDARA-MSPESSSDNKPPETLLLLK

*MAF5-AT5G65080.1* 0EKLLKEKNKVLASE---0VGKLKKILETGDERAVMSPENSSGHSPPETLPLLK

**Figure S7. Alignment of Arabidopsis FLC-clade members.** For each sequence in the alignment both its short and AtNumber (TAIR identifier) are provided. The positions of the introns with the coding region correspond to the aligned proteins are indicated using the characters 0, 1 or 2 which correspond to the phase of the intron. The aligned-positions containing the protein sequences encoded by the exons that are homologous to the 5’- exon of the mutually exclusive exon pair in *MAF1* (see main text) are underlined. The target introns are those introns that have been analyzed for the presence of sequences which are homologous to the 3'-exon of the mutually exclusive exon pair of *MAF1* (see main text).
