# Supplementary material for: Predicting the Impact of Alternative Splicing on Plant MADS Domain Protein Function
Source: PLoS One. 2012 Jan 25;7(1):e30524. doi: 10.1371/journal.pone.0030524 (PMC3266260; doi:10.1371/journal.pone.0030524)
Supplement: Figure S8 — Conservation of the MADS AFFECTING FLOWERING1 (MAF1) cryptic exon sequence. A. Multiple sequence alignment of the second intron from the Arabidopsis MAF1–3 genes. The conserved region corresponding to the 3′-cryptic exon from the mutually exclusive exon pair in the MAF1 gene is highlighted by the shaded box. B. Multiple sequence alignment of the translated intronic regions from the MAF2- and MAF3 gene and homologous 3′- cryptic exon of MAF1. (DOC) [file pone.0030524.s008.doc]

*MAF3* GTAAGT---TAGCTACGAACATCATC--AAAATTCTCTGGAATGCAGTTTTTGATGATAT

*MAF2* GTAAGT---TAGCTACGAATATCATT--AAAATTCTTCTGGATGCAGTTTTTGATGTTAT

*MAF1* GTAAGTAATTAGCTAAGAACGTCATTCTAATATTCTTCTGGATGCGGTTTTTGGTGTTAT

****** ****** *** **** ** ***** * **** ******* ** ***

*MAF3* TATGGGATAGAATTACTGGTCGAGCCTGAGATAACTCAATGATTTGAATTTCTTAAACTG

*MAF2* GAAGGGATAGAAGCACTGGTCGAACCTGAGATAACTCAATGTTTTGAATTTTTCGTACTG

*MAF1* GA-AGGATAGAAGCGCTGTTCAAGCCGGAGAAACCTCAATGTTTTGAACTCGTAACACCG

* ******** *** ** * ** **** * ******* ****** * * ** *

*MAF3* GACTTAATTTTCTTAAGTAACAGTTGTTGCATTTTTAGAAAAAACTCAAGAACTTTCACA

*MAF2* GACTTAATTCTCTCTAGTTACAGTTATTGCATTTTTCGGAAAA-----------CTCACA

*MAF1* AACTTAATTCTCTAGAGTTACAGTTATTGTGTCTACTGGAAAA--TACAAGAACTTCACA

******** *** *** ****** *** * * * **** *****

*MAF3* ATCTTTCTGACCATTGCCTTCGTTTTATCCATGTTCAG

*MAF2* ATCTTTCTGACCATTCCCTTCGTTTTCTCCATGTGCAG

*MAF1* ATCTTTCTGACCATTCC-----TTTTCTTCATGTGCAG

*************** * **** * ***** ***

*MAF3* IELLVEPEITQ*FEF

*MAF2* IEALVEPEITQCFEF

*MAF1* IEALFKPEKPQCFEL

**A**

**B**

**Figure S8. Conservation of the *MADS AFFECTING FLOWERING1 (MAF1)* cryptic exon sequence.**  **A.** Multiple sequence alignment of the second intron from the Arabidopsis *MAF1-3* genes. The conserved region corresponding to the 3’-cryptic exon from the mutually exclusive exon pair in the *MAF1* gene is highlighted by the shaded box. **B.** Multiple sequence alignment of the translated intronic regions from the *MAF2*- and *MAF3* gene and homologous 3’- cryptic exon of *MAF1*.
