# Supplementary material for: Predicting the Impact of Alternative Splicing on Plant MADS Domain Protein Function
Source: PLoS One. 2012 Jan 25;7(1):e30524. doi: 10.1371/journal.pone.0030524 (PMC3266260; doi:10.1371/journal.pone.0030524)
Supplement: Table S1 — MADS-box gene data. For each TAIR 10 locus that is annotated as a MADS-box gene it is indicated whether at least one predicted protein has: (i) an open reading frame fully supported by transcript data; (ii) experimental dimer interaction data, and (iii) a clear MIKC structure. It is further indicated whether a gene is alternatively (A) or constitutively spliced (C), whether a gene produces transcripts that are NMD targets and/or transcripts that are predicted to be translated into proteins. The number of inferred AS events and the annotated protein names are also given. (DOC) [file pone.0030524.s009.doc]

**Table S1. MADS-box gene data.**

For each TAIR 10 locus that is annotated as a MADS-box gene it is indicated whether at least one predicted protein has: (*i*) an open reading frame fully supported by transcript data; (*ii*) experimental dimer interaction data, and (*iii*) a clear MIKC structure. It is further indicated whether a gene is alternatively (A) or constitutively spliced (C), whether a gene produces transcripts that are NMD targets and/or transcripts that are predicted to be translated into proteins. The number of inferred AS events and the annotated protein names are also given.

| **TAIR 10 locus** | **Transcript support** | **Interaction data** | **MIKC** | **Splicing** | **NMD** | **Translatable** | **AS events** | **Annotation** |
| --- | --- | --- | --- | --- | --- | --- | --- | --- |
| AT1G01530 | + | + | - | C | - | + | - | AGL28 |
| AT1G17310 | + | - | - | C | - | + | - | AGL100 |
| AT1G18750 | + | + | - | A | + | + | 1 | AGL65 |
| AT1G22130 | + | + | - | C | - | + | - | AGL104 |
| AT1G22590 | + | + | - | C | - | + | - | AGL87 |
| AT1G24260 | + | + | + | A | - | + | 1 | AGL9, SEP3 |
| AT1G26310 | + | + | + | C | - | + | - | CAL1, AGL10, CAL |
| AT1G28450 | - | - | - | - | - | - | - | AGL58 |
| AT1G28460 | - | - | - | - | - | - | - | AGL59 |
| AT1G29960 | + | + | - | C | - | + | - | AGL64 |
| AT1G31140 | + | + | + | C | - | + | - | AGL63 |
| AT1G31630 | - | + | - | - | - | - | - | AGL86 |
| AT1G31640 | - | + | - | - | - | - | - | AGL92 |
| AT1G33070 | - | - | - | - | - | - | - | sequence-specific DNA binding |
| AT1G46408 | - | + | - | - | - | - | - | AGL97 |
| AT1G47760 | + | + | - | C | - | + | - | AGL102 |
| AT1G48150 | - | + | - | - | - | - | - | AGL74 |
| AT1G54760 | - | - | - | - | - | - | - | AGL85 |
| AT1G59810 | - | - | - | - | - | - | - | AGL50 |
| AT1G60040 | - | + | - | - | - | - | - | AGL49 |
| AT1G60880 | - | + | - | - | - | - | - | AGL56 |
| AT1G60920 | - | + | - | - | - | - | - | AGL55 |
| AT1G65300 | + | + | - | C | - | + | - | AGL38, PHE2 |
| AT1G65330 | - | + | - | - | - | - | - | AGL37, PHE1 |
| AT1G65360 | + | + | - | C | - | + | - | AGL23 |
| AT1G69120 | + | + | + | C | - | + | - | AGL7 |
| AT1G69540 | - | - | - | - | - | - | - | AGL94 |
| AT1G71692 | + | + | + | C | - | + | - | XAL1, AGL12 |
| AT1G72350 | - | - | - | - | - | - | - | AGL60 |
| AT1G77080 | + | + | + | A | + | + | 8 | FLM, AGL27, MAF1 |
| AT1G77950 | + | - | - | A | + | - | 1 | AGL67 |
| AT1G77980 | + | + | - | C | - | + | - | AGL66 |
| AT2G03060 | - | - | - | - | - | - | - | AGL30 |
| AT2G03710 | + | + | + | A | + | + | 2 | AGL3, SEP4 |
| AT2G11990 | - | - |  | - | - | - | - | - |
| AT2G14210 | + | + | + | C | - | + | - | AGL44, ANR1 |
| AT2G15660 | + | - | - | C | - | + | - | AGL95 |
| AT2G22540 | + | + | + | A | + | + | 2 | AGL22, SVP |
| AT2G22630 | + | + | + | C | - | + | - | AGL17 |
| AT2G24840 | + | - | - | C | - | + | - | AGL61 |
| AT2G26320 | - | - | - | - | - | - | - | AGL33 |
| AT2G26880 | - | - | - | - | - | - | - | AGL41 |
| AT2G28700 | + | - | - | C | - | + | - | AGL46 |
| AT2G34440 | + | - | - | C | - | + | - | AGL29 |
| AT2G40210 | - | + | - | - | - | - | - | AGL48 |
| AT2G42830 | + | + | + | A | - | + | 1 | AGL5, SHP2 |
| AT2G45650 | + | + | + | C | - | + | - | AGL6 |
| AT2G45660 | + | + | + | C | - | + | - | SOC1, AGL20 |
| AT3G02310 | + | + | + | C | - | + | - | AGL4, SEP2 |
| AT3G04100 | + | - | - | C | - | + | - | AGL57 |
| AT3G05860 | + | + | - | A | - | + | 1 | AGL45 |
| AT3G18650 | - | + | - | - | - | - | - | AGL103 |
| AT3G30260 | + | - | + | C | - | + | - | AGL79 |
| AT3G54340 | + | - | + | A | - | + | 2 | AP3 |
| AT3G57230 | + | + | + | A | + | + | 1 | AGL16 |
| AT3G57390 | + | - | + | A | + | + | 2 | AGL18 |
| AT3G58780 | + | + | + | A | - | + | 1 | AGL1, SHP1 |
| AT3G61120 | + | + | + | C | - | + | - | AGL13 |
| AT3G66656 | + | - | - | C | - | + | - | AGL91 |
| AT4G02235 | + | - | - | A | - | + | 1 | AGL51 |
| AT4G02240 | - | - |  | - | - | - | - | - |
| AT4G09960 | + | + | + | A | - | + | 1 | AGL11, STK |
| AT4G11250 | + | + | - | C | - | + | - | AGL52 |
| AT4G11880 | + | + | + | C | - | + | - | AGL14 |
| AT4G18960 | + | + | + | C | - | + | - | AG |
| AT4G22950 | + | + | + | C | - | + | - | AGL19 |
| AT4G24540 | + | + | + | C | - | + | - | AGL24 |
| AT4G36590 | + | + | - | C | - | + | - | AGL40 |
| AT4G37940 | + | + | + | C | - | + | - | AGL21 |
| AT5G04640 | + | + | - | C | - | + | - | AGL99 |
| AT5G06500 | + | + | - | C | - | + | - | AGL96 |
| AT5G10140 | + | - | + | A | - | + | 13 | FLF, AGL25, FLC |
| AT5G13790 | + | + | + | A | + | + | 2 | AGL15 |
| AT5G15800 | + | + | + | C | - | + | - | AGL2, SEP1 |
| AT5G20240 | + | - | + | C | - | + | - | PI |
| AT5G23260 | + | + | + | A | - | + | 1 | AGL32, ABS, TT16 |
| AT5G26575 | - | - |  | - | - | - | - | - |
| AT5G26580 | + | - | - | C | - | + | - | AGL34 |
| AT5G26630 | - | - | - | - | - | - | - | AGL35 |
| AT5G26650 | + | + | - | C | - | + | - | AGL36 |
| AT5G26870 | + | + | - | A | + | + | 1 | AGL26 |
| AT5G26950 | + | - | - | C | - | + | - | AGL93 |
| AT5G27050 | + | + | - | C | - | + | - | AGL101 |
| AT5G27070 | + | + | - | C | - | + | - | AGL53 |
| AT5G27090 | + | + | - | C | - | + | - | AGL54 |
| AT5G27130 | - | + | - | - | - | - | - | AGL39 |
| AT5G27580 | + | + | - | C | - | + | - | AGL89 |
| AT5G27960 | + | + | - | A | - | + | 1 | AGL90 |
| AT5G37420 | - | - | - | - | - | - | - | AGL105 |
| AT5G38620 | + | + | - | C | - | + | - | AGL73 |
| AT5G38740 | - | + | - | - | - | - | - | AGL77 |
| AT5G39750 | + | - | - | C | - | + | - | EMB3008, AGL81 |
| AT5G39810 | - | - | - | - | - | - | - | AGL98 |
| AT5G40120 | + | + | - | C | - | + | - | AGL76 |
| AT5G40220 | + | + | - | C | - | + | - | AGL43 |
| AT5G41200 | + | - | - | C | - | + | - | AGL75 |
| AT5G48670 | + | + | - | C | - | + | - | FEM111, AGL80 |
| AT5G49420 | + | + | - | C | - | + | - | AGL84 |
| AT5G49490 | - | + | - | - | - | - | - | AGL83 |
| AT5G51860 | + | + | + | A | - | + | 1 | AGL72 |
| AT5G51870 | + | + | + | C | - | + | - | AGL71 |
| AT5G55690 | + | - | - | C | - | + | - | AGL47 |
| AT5G58890 | - | + | - | - | - | - | - | AGL82 |
| AT5G60440 | + | + | - | A | - | + | 1 | AGL62 |
| AT5G60910 | + | + | + | C | - | + | - | FUL, AGL8 |
| AT5G62165 | + | + | + | A | - | + | 5 | AGL42 |
| AT5G65050 | + | - | + | A | + | + | 5 | MAF2, AGL31 |
| AT5G65060 | + | - | + | A | + | + | 6 | AGL70, FCL3, MAF3 |
| AT5G65070 | - | - | - | - | - | - | - | AGL69, FCL4, MAF4 |
| AT5G65080 | + | - | + | C | - | + | - | MAF5, AGL68 |
| AT5G65330 | + | + | - | C | - | + | - | AGL78 |
