# Supplementary material for: Predicting the Impact of Alternative Splicing on Plant MADS Domain Protein Function
Source: PLoS One. 2012 Jan 25;7(1):e30524. doi: 10.1371/journal.pone.0030524 (PMC3266260; doi:10.1371/journal.pone.0030524)
Supplement: Table S2 — Effect of AS on predicted interaction motifs. The residues from each motif that overlap with AIPs are red. Residues that are inserted into motifs are surrounded by square brackets. Multiple overlapping interaction motifs that are affected by the same AIP are stacked. Multiple non-overlapping motifs that are affected by the same AS event are numbered. (DOC) [file pone.0030524.s010.doc]

**Table S2. Effect of AS on predicted interaction motifs.** The residues from each motif that overlap with AIPs are red. Residues that are inserted into motifs are surrounded by square brackets. Multiple overlapping interaction motifs that are affected by the same AIP are stacked. Multiple non-overlapping motifs that are affected by the same AS event are numbered.

| **TAIR 10 Locus** | **Symbol** | **Event** | **Description motif effect** | **Isoform containing the affected motif** |
| --- | --- | --- | --- | --- |
| AT1G77080 | MAF1/FLM | intron retention | Motif 1:  lEtvQrlA  Motif 2:  LpSSSdkk | Motif 1: *MAF1.4*  Motif 2: Retained intron |
|  |  | mutually exclusive exons | Motif 1:  kIIDRYei  Motif 2:  elraLdLE  Motif 3:  QcfeLdLE | Motif 1: *MAF1.4*  Matif 2: *MAF1.2*  Motif3: *MAF1.2* |
| AT2G22540 | SVP | alternative donor | LFEFcSss  EFCSSsmk  FcSSsMke  CSSSmkev  sSsMkeVL  SSMkevLE | *SVP3* |
| AT2G42830 | SHP2 | alternative donor | QkR[VK]EieLq | *SHP-2.2* |
| AT3G58780 | SHP1 | alternative acceptor | GTIERYkk | *SHP-1.1* |
| AT4G09960 | STK | exon skipping | Motif 1:  RsKKhElL  kkhellLV  lLveIenA  Motif 2:  QkREieLd | *STK.1* |
| AT5G23260 | ABS-1, ABS-2 | alternative acceptor | ReRKnElM | *ABS.2* |
