# Supplementary material for: Predicting the Impact of Alternative Splicing on Plant MADS Domain Protein Function
Source: PLoS One. 2012 Jan 25;7(1):e30524. doi: 10.1371/journal.pone.0030524 (PMC3266260; doi:10.1371/journal.pone.0030524)
Supplement: Table S3 — Sequences of oligonucleotides used in this study. The first listed oligonucleotide is always the forward primer and the second one the reverse. (DOC) [file pone.0030524.s011.doc]

**Table S3. Sequences of oligonucleotides used in this study.** The first listed oligonucleotide is always the forward primer and the second one the reverse.

| **TAIR locus** | **Symbol** | **Oligonucleotides** |
| --- | --- | --- |
| AT5G08290 | *YLS8* (Reference gene) | PDS4009 (TTACTGTTTCGGTTGTTCTCCATTT)  PDS4010 (CACTGAATCATGTTCGAAGCAAGT) |
| AT2G22540 | *SVP1/SVP3*^1)^ | PDS3106 (GAAGAGAACGAGCGACTTGG)  PDS3107 (GAGCTCTCGGAGTCAACAGG) |
| AT2G22540 | *SVP3* | PDS2264 (ACCGGAAAACTGTTCGACATGA)  PDS2265 (TTCTTTACTCATTCGGGCGTGAT) |
| AT1G77080 | *MAF1.2* | PDS4470 (CCTCAATGTTTTGAACTCGATC)  PDS4471 (TCGACATTTGGTTCTTCAAGCTTGC) |
| AT1G77080 | *MAF1.4* | PDS4474 (GATCGTTATGAAATACAACATGC)  PDS4475 (GTATTCTTTCCCATCTGGCTAGC) |
| AT5G65050 | *MAF2.2* | PDS3124 (AGCTCGAGACTGCTCTGTCC)  PDS3125 (TCAACTGATGAATTAGCTTCAAGA) |
| [AT3G58780](http://www.arabidopsis.org/servlets/TairObject?id=39912&type=locus) | *SHP1.1* | PDS4644 (CAGTGTGAGGGGTACAATTG)  PDS4645 (AAGTGATTCCCCAACAATATGC) |
| [AT3G58780](http://www.arabidopsis.org/servlets/TairObject?id=39912&type=locus) | *SHP1.2* | PDS4646 (CAGGTACAAGAAAGCTTGTTC)  PDS4645 (AAGTGATTCCCCAACAATATGC) |
| AT2G42830 | *SHP2.1* | PDS4650 (GGGAAATCGAGCTGCAAAACG)  PDS3071 (CAGAGGTGGTTGGTCTTGGT) |
| AT2G42830 | *SHP2.2* | PDS4651 (GGGTAAAAGAAATCGAGCTGC)  PDS3071 (CAGAGGTGGTTGGTCTTGGT) |
| AT4G09960 | *STK.1* | PDS3082 (CGCAGAAAAGGGAGATTGAG)  PDS3083 (CCAGATCCAGAACCAGCAGT) |
| AT4G09960 | *STK.2* | PDS4652 (GGTCCAAGAAGGAGATTGAGC)  PDS3083 (CCAGATCCAGAACCAGCAGT) |
| AT5G23260 | *ABS.1* | PDS4656 (GAGCGTAAGCAACAGTTGGA)  PDS4657 (GTTGTTGTTGCTGCTGCTCT) |
| AT5G23260 | *ABS.2* | PDS3130 (AGCGTAAGAATGAGTTAATGC)  PDS4657 (GTTGTTGTTGCTGCTGCTCT) |
| AT1G24260 | *SEP3.1* | PDS4658 (CCTTAGCAGAACTTAGTAGCC)  PDS4659 (CAGTCAGCATGCGTTCCTTA) |
| AT1G24260 | *SEP3.2* | PDS4660 (GCAGTTGAACTTAGTAGCCAG)  PDS4659 (CAGTCAGCATGCGTTCCTTA) |

1. No oligonucleotides could be designed that are specific for the *SVP1* splicing variant only. The indicated primers detect both *SVP1* and *SVP3.*
